# Electricity in Exciting Uterine Action during Delivery

**Published:** 1846-02

**Authors:** 


*On the Use of Electricity in Exciting Uterine Action during Delivery.*—I was requested to see Mary Cook, æt. 39 years, in her sixteenth confinement, on Friday morning the 16th of June.

On my arrival at the house, I learned that her previous labors had been tolerably good, with two or three exceptions, when they had been considerably protracted for want of pains. Her health has always been delicate, and for the last few weeks she has had a troublesome cough, attended with copious expectoration; emaciation and occasional night-sweats—symptoms which naturally led to the opinion that she was suffering from phthisis, although subsequently this diagnosis was not confirmed by a physical examination of the chest. On the Sunday evening prior to my visiting her, she was first attacked with premonitory labor, soon succeeded by regular and frequent pains, which, on the following morning, abated, but never entirely left her till Wednesday night, when the liquor amnii was discharged.

At 1 A.M. on the Friday, the pains returned with considerable vigor, but did not last above an hour, and at 6 o'clock were again renewed for a short time. It was about four hours after this period that I found Mr. T. with the patient, to whom he had given a dose of the tincture of ergot, and also some spirit and water; but these measures were followed by only a few slight and ineffectual pains.

On making an examination, I found the vagina freely lubricated, the os uteri dilated, the head of the child small, presenting in the right oblique diameter, and in the pelvic cavity; in fact, there appeared no obstacle whatever to the completion of the case but uterine inertia, which I considered was owing to constitutional debility, arising chiefly from the state of the chest. She had rather an anxious countenance, a small and frequent pulse; complained of great thirst and languor, and of having had no sleep for several nights.

It was obvious that if uterine contraction could not be somehow induced, and it must be remembered that the ergot had been already tried, the alternative would be instrumental delivery; and this, considering the weak state of the patient's health, and the most improbable and unpleasant result of hemorrhage from atony of the womb was not desirable.

At the suggestion of Dr. Lever, who kindly lent me his electro-galvanic apparatus, I resolved on a fair trial of galvanism, and accordingly, with my friend, Mr. Richardson, proceeded to its application externally and obliquely across the anterior surface of the uterus. In a few minutes the effect was apparent. Regular, strong, frequent pains came on, and in a quarter of an hour from the first application of the remedy, a living male child and placenta was expelled, attended with the least degree of hemorrhage I ever witnessed.

The uterus was immediately firmly and permanently contracted, and, with the exception of slight soreness of the abdomen, the patient expresses herself as quite comfortable, and, up to the present time, setting aside debility, she has progressed favorably.—*Med. Gaz.*—*Ibid.*

